# Supplementary material for: Short-Term and Long-Term Carcinogenic Effects of Food Contaminants (4-Hydroxynonenal and Pesticides) on Colorectal Human Cells: Involvement of Genotoxic and Non-Genomic Mechanisms
Source: Cancers (Basel). 2021 Aug 27;13(17):4337. doi: 10.3390/cancers13174337 (PMC8431687; doi:10.3390/cancers13174337)
Supplement: Supplementary file 1 [file cancers-13-04337-s001.zip › cancers-1307501-supplementary.pdf]

cancers

MDPI

Supplementary Files

Short-Term and Long-Term Carcinogenic Effects of Food Contaminants (4-Hydroxynonenal and Pesticides) On Colorectal Human Cells: Involvement of Genotoxic and Non-Genomic Mechanisms

Liana C. Arnaud, Thierry Gauthier, Augustin Le Naour, Saleha Hashim, Nathalie Naud, Jerry W Shay, Fabrice H. Pierre, Elisa Boutet-Robinet and Laurence Huc

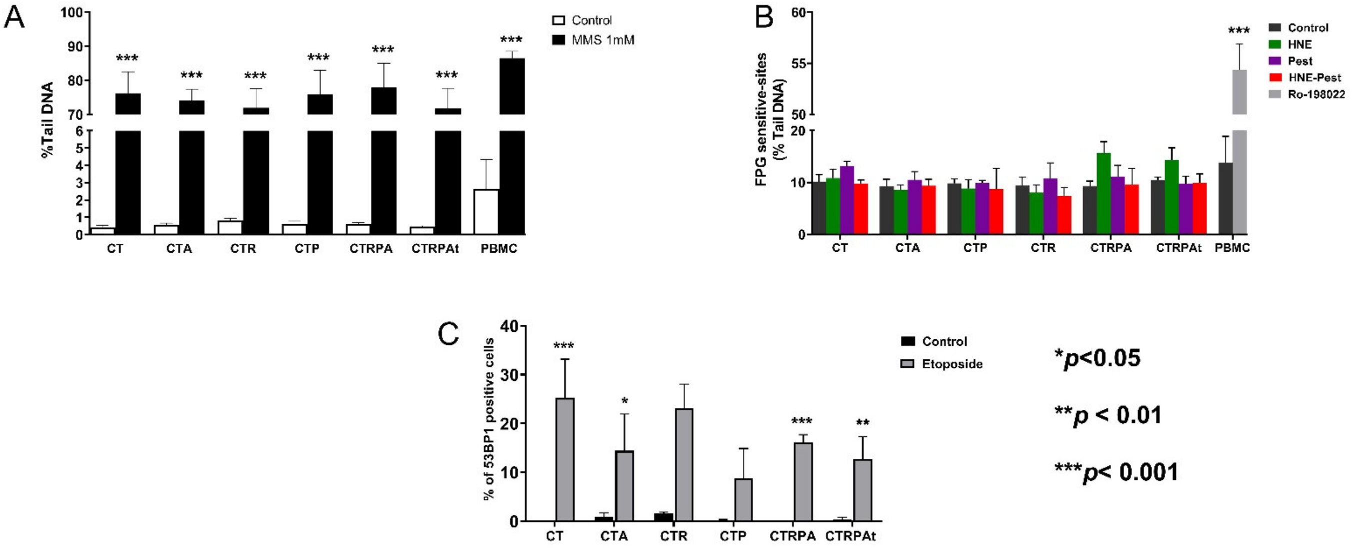

Figure S1. Positive controls for genotoxicity assays. (**A**) DNA damage evaluated by alkaline comet assay at 2h post-treatment of MMS (positive control) in HCECs and PBMC, an internal standard from a cryopreserved batch as recommended by Minimum Information for Reporting Comet Assay (MIRCA) (Møller et al., 2020). (**B**) Quantification of Fpg-sensitive sites using Fpg modified comet assay in HCECs at 2h post-treatment and in PBMC treated or not with Ro 19-8022 (positive control). FPG sensitive sites were calculated as described in material and method section. (**C**) Quantification of HCECs positives for 53BP1 after 2h of treatment to etoposide (positive control). All the results (A, B and C) represent the mean ± SEM of three independent experiments; statistical differences were analysed by post-hoc tests of a 2-way ANOVA model between control and treated cells (* p < 0.05, ** *p* < 0.01, *** *p* < 0.001).

Cell Titer Glo® Acute exposure	
Effect	*P*-value
Cell line	$p < 0.001$ (***)
Treatment	$p = 0.794$ (ns)
Cell line*Treatment	$p > 0.999$ (ns)

Xcelligence® Acute exposure	
Effect	*P*-value
Cell line	$p = 0.007$ (**)
Treatment	$p = 0.995$ (ns)
Cell line*Treatment	$p > 0.999$ (ns)

Alkaline comet assay Acute exposure	
Effect	*P*-value
Cell line	$p = 0.812$ (ns)
Treatment	$p < 0.001$ (***)
Cell line*Treatment	$p = 0.997$ (ns)

Fpg-comet assay Acute exposure	
Effect	*P*-value
Cell line	$p = 0.762$ (ns)
Treatment	$p < 0.001$ (***)
Cell line*Treatment	$p = 0.994$ (ns)

53BP1 immunostaining Acute exposure	
Effect	*P*-value
Cell line	$p = 0.002$ (**)
Treatment	$p < 0.001$ (***)
Cell line*Treatment	$p = 0.021$ (*)

Counting cells Long-term exposure	
Effect	*P*-value
Cell line	$p < 0.001$ (***)
Treatment	$p < 0.001$ (***)
Cell line*Treatment	$p = 0.005$ (**)

Soft agar assay Long-term exposure	
Effect	*P*-value
Cell line	$p < 0.001$ (***)
Treatment	$p < 0.001$ (***)
Cell line*Treatment	$p < 0.001$ (***)

γH2AX immunostaining Long-term exposure	
Effect	*P*-value
Cell line	$p = 0.301$ (ns)
Treatment	$p < 0.001$ (***)
Cell line*Treatment	$p = 0.747$ (ns)

53BP1 immunostaining Long-term exposure	
Effect	*P*-value
Cell line	$p = 0.190$ (ns)
Treatment	$p = 0.697$ (ns)
Cell line*Treatment	$p = 0.054$ (*)

Basal OCR Seahorse® Long-term exposure	
Effect	*P*-value
Cell line	$p = 0.136$ (ns)
Treatment	$p = 0.003$ (**)
Cell line*Treatment	$p = 0.304$ (ns)

Basal ECAR Seahorse® Long-term exposure	
Effect	*P*-value
Cell line	$p = 0.143$ (ns)
Treatment	$p = 0.003$ (**)
Cell line*Treatment	$p = 0.265$ (ns)

Maximal OCR Seahorse® Long-term exposure	
Effect	*P*-value
Cell line	$p = 0.013$ (*)
Treatment	$p < 0.001$ (***)
Cell line*Treatment	$p = 0.166$ (ns)

ATP production Seahorse® Long-term exposure	
Effect	*P*-value
Cell line	$p < 0.001$ (***)
Treatment	$p = 0.002$ (**)
Cell line*Treatment	$p = 0.013$ (*)

E-cadherin immunoblots Long-term exposure	
Effect	*P*-value
Cell line	$p = 0.222$ (ns)
Treatment	$p = 0.097$ (*)
Cell line*Treatment	$p = 0.833$ (ns)

Vimentin immunoblots Long-term exposure	
Effect	*P*-value
Cell line	$p = 0.818$ (ns)
Treatment	$p = 0.198$ (ns)
Cell line*Treatment	$p = 0.915$ (ns)

Figure S2. Summarized tables of statistical analysis.

Cell Titer Glo® Acute exposure	
Effect	P-value
Cell line	$p > 0.999$ (ns)
Treatment	$p = 0.794$ (ns)
Cell line*Treatment	$p > 0.999$ (ns)

Xcelligence® Acute exposure	
Effect	P-value
Cell line	$p = 0.997$ (ns)
Treatment	$p = 0.995$ (ns)
Cell line*Treatment	$p > 0.999$ (ns)

Alkaline comet assay Acute exposure	
Effect	P-value
Cell line	$p = 0.152$ (ns)
Treatment	$p < 0.001$ (***)
Cell line*Treatment	$p = 0.997$ (ns)

Fpg-comet assay Acute exposure	
Effect	P-value
Cell line	$p = 0.881$ (ns)
Treatment	$p < 0.001$ (***)
Cell line*Treatment	$p = 0.994$ (ns)

53BP1 immunostaining Acute exposure	
Effect	P-value
Cell line	$p < 0.001$ (***)
Treatment	$p < 0.001$ (***)
Cell line*Treatment	$p = 0.021$ (*)

Counting cells Long-term exposure	
Effect	P-value
Cell line	$p = 0.168$ (ns)
Treatment	$p < 0.001$ (***)
Cell line*Treatment	$p = 0.326$ (ns)

Soft agar assay Long-term exposure	
Effect	P-value
Cell line	$p < 0.001$ (***)
Treatment	$p < 0.001$ (***)
Cell line*Treatment	$p < 0.001$ (***)

γH2AX immunostaining Long-term exposure	
Effect	P-value
Cell line	$p = 0.213$ (ns)
Treatment	$p < 0.001$ (***)
Cell line*Treatment	$p = 0.747$ (ns)

53BP1 immunostaining Long-term exposure	
Effect	P-value
Cell line	$p < 0.001$ (***)
Treatment	$p = 0.658$ (ns)
Cell line*Treatment	$p = 0.044$ (*)

Basal OCR Seahorse® Long-term exposure	
Effect	P-value
Cell line	$p = 0.142$ (ns)
Treatment	$p = 0.003$ (**)
Cell line*Treatment	$p = 0.304$ (ns)

Basal ECAR Seahorse® Long-term exposure	
Effect	P-value
Cell line	$p = 0.172$ (ns)
Treatment	$p = 0.003$ (**)
Cell line*Treatment	$p = 0.265$ (ns)

Maximal OCR Seahorse® Long-term exposure	
Effect	P-value
Cell line	$p = 0.059$ (*)
Treatment	$p < 0.001$ (***)
Cell line*Treatment	$p = 0.166$ (ns)

ATP production Seahorse® Long-term exposure	
Effect	P-value
Cell line	$p < 0.001$ (***)
Treatment	$p = 0.002$ (**)
Cell line*Treatment	$p = 0.013$ (*)

E-cadherin immunoblots Long-term exposure	
Effect	P-value
Cell line	$p = 0.072$ (*)
Treatment	$p = 0.097$ (*)
Cell line*Treatment	$p = 0.833$ (ns)

Vimentin immunoblots Long-term exposure	
Effect	P-value
Cell line	$p = 0.053$ (*)
Treatment	$p = 0.198$ (ns)
Cell line*Treatment	$p = 0.915$ (ns)

Figure S3. Summarized tables of statistical refocused data.

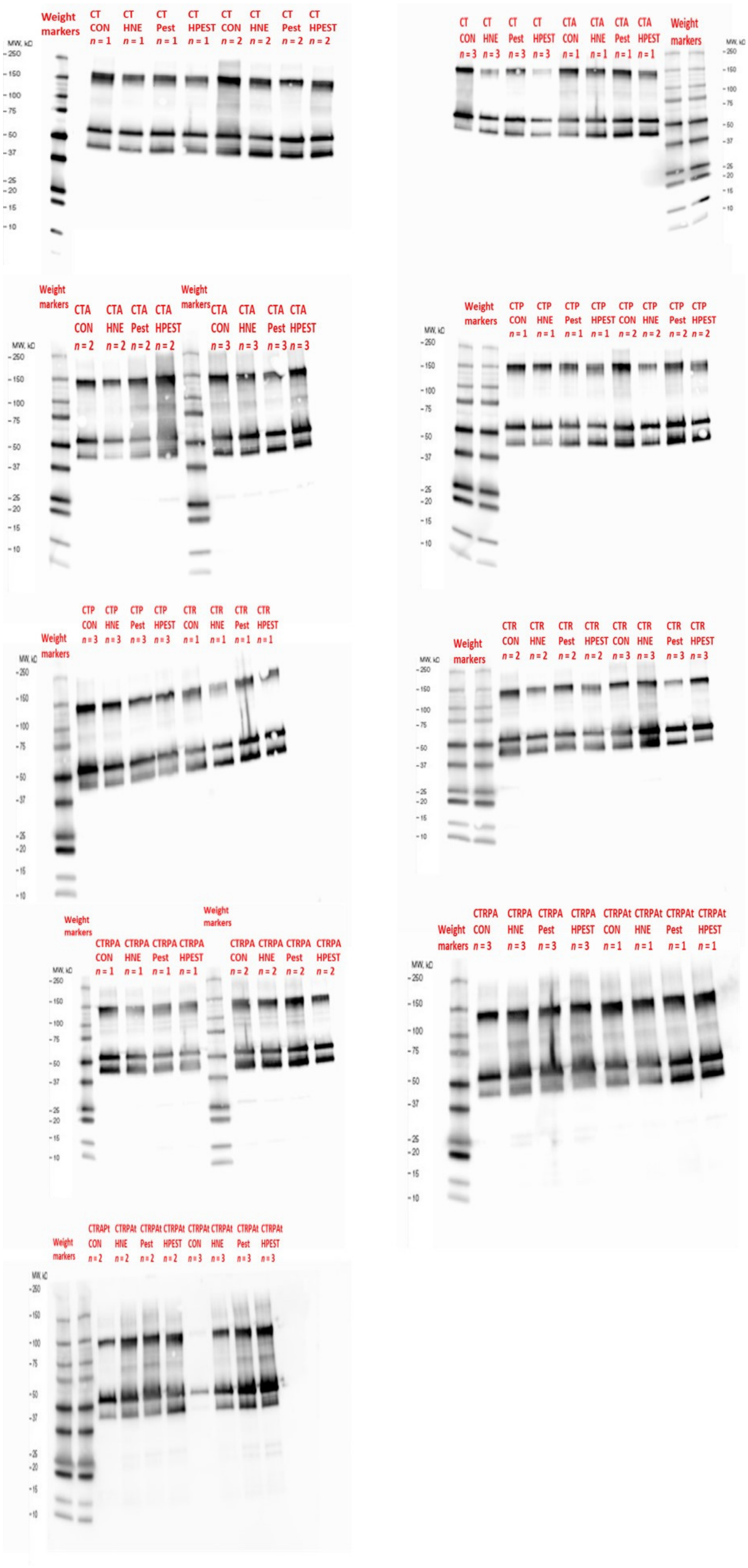

Figure S4. Western blot images.
